# Observation of the blood pressure changes in preterm infants with gestational age of 28 to <37 weeks within 7 days after birth: a single center retrospective study

**DOI:** 10.3389/fped.2026.1757399

**Published:** 2026-03-09

**Authors:** Yuqiang Guan, Lei Li, Wenhui Bie, Honglin Sun, Peiru Yu

**Affiliations:** Department of Cardiology, Children’s Hospital Affiliated to Shandong University, Jinan, China

**Keywords:** blood preasure, first 7 days after birth, gestational age of 28 to <37 weeks, preterm infants, regularity of blood pressure changes

## Abstract

**Objective:**

To monitor the regularity of blood pressure changes in preterm infants with a gestational age of 28 to <37 weeks within 7 days after birth.

**Method:**

A retrospective analysis was conducted on preterm infants who were hospitalized in the Department of Neonatology of our hospital from January 2022 to January 2025, received non-invasive arterial blood pressure monitoring, and did not receive treatment for hypotension. Basic information of preterm infants (with a gestational age of 28 to <37 weeks) and their mothers, blood pressure data within 7 days after birth, clinical outcomes, and complication status were collected. The blood pressure variation trend of preterm infants (with a gestational age of 28 to <37 weeks) without hypotension treatment was analyzed to provide data support for establishing the blood pressure reference ranges for preterm infants in different gestational age subgroups.

**Results:**

A total of 191 preterm infants with a gestational age of 28 to <37 weeks were included. Both systolic blood pressure (SBP) and mean arterial pressure (MAP) demonstrated an upward trend within 7 days after birth (*P* < 0.001). For SBP: There were significant differences between the 1st postnatal day and the 3rd to 7th postnatal days, as well as between the 2nd postnatal day and the 6th postnatal day. No significant differences in SBP were observed between other time points. For MAP: Significant differences were noted between the 1st postnatal day and the 5th to 6th postnatal days, and between the 2nd postnatal day and the 6th postnatal day. No significant differences in MAP were found between other time points. No obvious variation trend was observed in diastolic blood pressure (DBP) within 1 week after birth.

**Conclusion:**

Within 7 days after birth, SBP and MAP of preterm infants with a gestational age of 28 to <37 weeks show a spontaneous upward trend with the increase of postnatal age, among which the increase of SBP is more obvious and tends to stabilize about 3 days after birth. The postnatal blood pressure reference ranges vary among preterm infants of different gestational ages, which provides a reference basis for the early postnatal blood pressure assessment and clinical management of preterm infants with different gestational ages.

## 1 Introduction

In China, the preterm birth rate has been increasing at a pace of more than 1% per year (1). During the transition from intrauterine fetal circulation to postnatal neonatal circulation, preterm infants are prone to hemodynamic instability and hypotension due to factors such as immature development of cardiomyocytes, changes in preload and afterload, and frequently complicated by delayed closure of the ductus arteriosus, perinatal asphyxia, infection, and adrenal insufficiency (2, 3). These conditions can further lead to severe complications such as intraventricular hemorrhage (IVH), necrotizing enterocolitis (NEC), and renal failure (4). Existing studies have demonstrated that blood pressure in preterm infants is closely associated with gestational age at birth and postnatal age (5). However, most research has focused on extremely preterm infants (<28 weeks) (6), leaving the broader population of moderate to late preterm infants with a gestational age of 28 to <37 weeks relatively understudied and lacking longitudinal data on early postnatal blood pressure changes. Therefore, this study retrospectively analyzed blood pressure monitoring data of preterm infants (with a gestational age of 28 to <37 weeks) admitted to the Department of Neonatology, Children's Hospital Affiliated to Shandong University from January 2022 to January 2025. The aim was to observe blood pressure levels and their dynamic variation trends within 7 days after birth in this population, so as to provide a reference basis for clinical blood pressure management of preterm infants in this gestational age range.

## 2 Subjects and methods

### 2.1 Study subjects

This study included preterm infants with a gestational age of 28 to <37 weeks who were admitted to the Department of Neonatology, Children's Hospital Affiliated to Shandong University from January 2022 to January 2025. Inclusion Criteria: (1) Gestational age at birth between 28 weeks and <37 weeks; (2) Admitted to the Neonatology Department of our hospital for treatment within 1 day after birth; (3) Received non-invasive blood pressure monitoring using upper limb arterial blood pressure (brachial artery) after admission; (4) Duration of blood pressure monitoring ≥7 days.To eliminate the confounding effects of medications, incomplete data, and hemodynamically unstable conditions on blood pressure measurement and interpretation, and to ensure the homogeneity of the study population, the following exclusion criteria were applied: (1) Received treatment for hypotension within 7 days after birth; (2) Incomplete clinical data (including missing general information of the infant or missing blood pressure data at key time points); (3) Received treatment for hypertension (including furosemide, spironolactone, and hydrochlorothiazide); (4) Complicated with major diseases affecting blood pressure (including heart failure caused by large patent ductus arteriosus [L-PDA] (7), pulmonary hypertension [PH], ventricular septal defect [VSD], etc.); (5) Complicated with sepsis.This study has been approved by the Ethics Committee of Children's Hospital Affiliated to Shandong University.

### 2.2 Study methods

1. Blood Pressure Monitoring: Peripheral arterial blood pressure monitoring was performed using a GE Dash 3000 multiparameter monitor. Monitoring indicators included systolic blood pressure (SBP) and diastolic blood pressure (DBP). Mean arterial pressure (MAP) was calculated by data collectors using the formula: MAP = DBP + 1/3(SBP − DBP). The cuff oscillometric method was used for measurement, with the ratio of cuff width to arm circumference mentioned as close to 0.50 as possible (8), and the cuff was required to cover 80% of the upper arm length. Measurement time was fixed from 9:00 AM to 5:00 PM daily. The infant was in a supine position during measurement, and the brachial artery of the right upper arm was fixed as the measurement site. Measurements were performed in a quiet environment, 1–2 h after the infant's feeding, and when the infant was in a sleep or quiet awake state. The daily blood pressure value of each infant was the average of at least 3 independent measurements, with an interval of more than 2 min between each measurement.All measurements were performed by uniformly trained neonatal nursing staff in strict accordance with the aforementioned standardized protocol to ensure consistency in measurement procedures and cuff usage.
2. Definition Criteria for Hypotension Treatment: Receipt of any of the following interventions within 7 days after birth was defined as hypotension treatment: (1) Vasoactive drugs: dopamine, dobutamine; (2) Adrenocortical hormones: hydrocortisone, methylprednisolone, dexamethasone; (3) Volume resuscitation: normal saline volume expansion; (4) Circulatory support: blood transfusion therapy for hypovolemia.
3. Data Collection: Two trained researchers extracted medical records using independent double entry and cross-verification. The collected content included: (1) General Information: Gestational age, gender, birth weight, mode of delivery, 1-minute Apgar score after birth, presence of patent ductus arteriosus (PDA), history of hypotension treatment within the first 7 days after birth; maternal gestational complications (gestational hypertension, eclampsia, intrapartum infection) and maternal medication history during pregnancy (dexamethasone, antihypertensive drugs (including labetalol and nifedipine), magnesium sulfate; (2) Blood Pressure Data: Daily monitored values of SBP, DBP, and MAP from 1 to 7 days after birth(All blood pressure measurement devices were regularly calibrated in accordance with departmental specifications. Cuff sizes were selected based on the infant's upper arm circumference, and cuff sizes remained consistent for the same infant during measurement to reduce measurement bias. All blood pressure data were manually verified one by one to ensure data integrity and accuracy. For a small number of missing blood pressure measurements, only the available valid values were used for analysis, and no data imputation was performed); (3) Clinical Outcomes and Complications: Discharge outcome of the infant; occurrence and grading of preterm complications, including bronchopulmonary dysplasia (BPD), intraventricular hemorrhage (IVH), and retinopathy of prematurity (ROP).

### 2.3 Statistical methods

SPSS 25.0 and Graphpad 8.0 statistical software were used for data analysis and graphing. Count data were expressed as cases (%). Measurement data were tested for normality using the Shapiro–Wilk test: normally distributed data were described as mean ± standard deviation ($\bar{x}\pm s$), and non-normally distributed data were described as median (25th percentile, 75th percentile) [M (P25, P75)]. The Friedman M test was used for statistical analysis of blood pressure data from 1 to 7 days after birth; if there was a statistical difference, Bonferroni correction was further used for *post-hoc* pairwise comparisons. Blood pressure values of different gestational ages were expressed as P2.5–P97.5 (P50) (This interval represents 95% of the data distribution and comprehensively reflects the physiological fluctuation range of blood pressure in the vast majority of preterm infants). *P* < 0.05 was considered statistically significant.

## 3 Results

### 3.1 General information

A total of 404 preterm infants with a gestational age of 28 to <37 weeks were admitted during the study period. Eighty-four infants with incomplete non-invasive blood pressure monitoring data within 7 days and 129 infants who received hypotension treatment within 7 days were excluded, resulting in a final sample of 191 infants. Among them, there were 110 males and 81 females; the gestational age was 33.85 (31.82, 35.32) weeks, including 49 infants with 28 ≤ gestational age <32 weeks, 49 infants with 32 ≤ gestational age <34 weeks, and 93 infants with 34 ≤ gestational age <37 weeks; the birth weight was (2.07 ± 0.64) kg; the 1-minute Apgar score was 9.0 (8.0, 10.0) points; 60 infants had patent ductus arteriosus (PDA); 30 infants were delivered by cesarean section; 60 infants received invasive mechanical ventilation; 36 mothers had gestational hypertension; 9 mothers had gestational eclampsia; 20 mothers had gestational diabetes mellitus (GDM) and 32 had infections; 6 mothers used antihypertensive drugs prenatally, 20 used magnesium sulfate, and 92 used dexamethasone.A total of 4,011 non-invasive blood pressure measurements were performed within 7 days after birth, with a mean of 3 measurements per infant per day and an interval of ≥2 min between consecutive measurements, as detailed in Table 1.

**Table 1:** General information of preterm infants and pregnancy information of their mother.

Maternal and neonatal characteristics	*n* (%)/Mean ± SD/ Median (IQR)
Included sample size	191
Male/Female	110/81 (57.6/42.4)
Birth weight (kg)	2.07 ± 0.64
Gestational age (weeks)	33.85 (31.82,35.32)
28 ≤ gestational age <32 weeks	49 (25.7)
32 ≤ gestational age <34 weeks	49 (25.7)
34 ≤ gestational age <37 weeks	93 (48.6)
1 min Apgar score	9.0 (8.0,10.0)
Patent ductus arteriosus (PDA)	60 (31.4)
Cesarean section	30 (15.7)
Invasive mechanical ventilation	60 (31.4)
Gestational hypertension	36 (18.8)
Gestational eclampsia	9 (4.7)
Gestational diabetes mellitus (GDM)	20 (10.4)
Gestational infection	32 (16.7)
Antihypertensive drugs administered prenatally	6 (3.0)
Labetalol	4 (2.0)
Nifedipine	2 (1.0)
Magnesium sulfate administered prenatally	20 (10.4)
Dexamethasone administered prenatally	92 (48.1)

### 3.2 Blood pressure variation trend of preterm infants with gestational Age of 28 to <37 weeks within 7 days after birth

**Figure 1:**
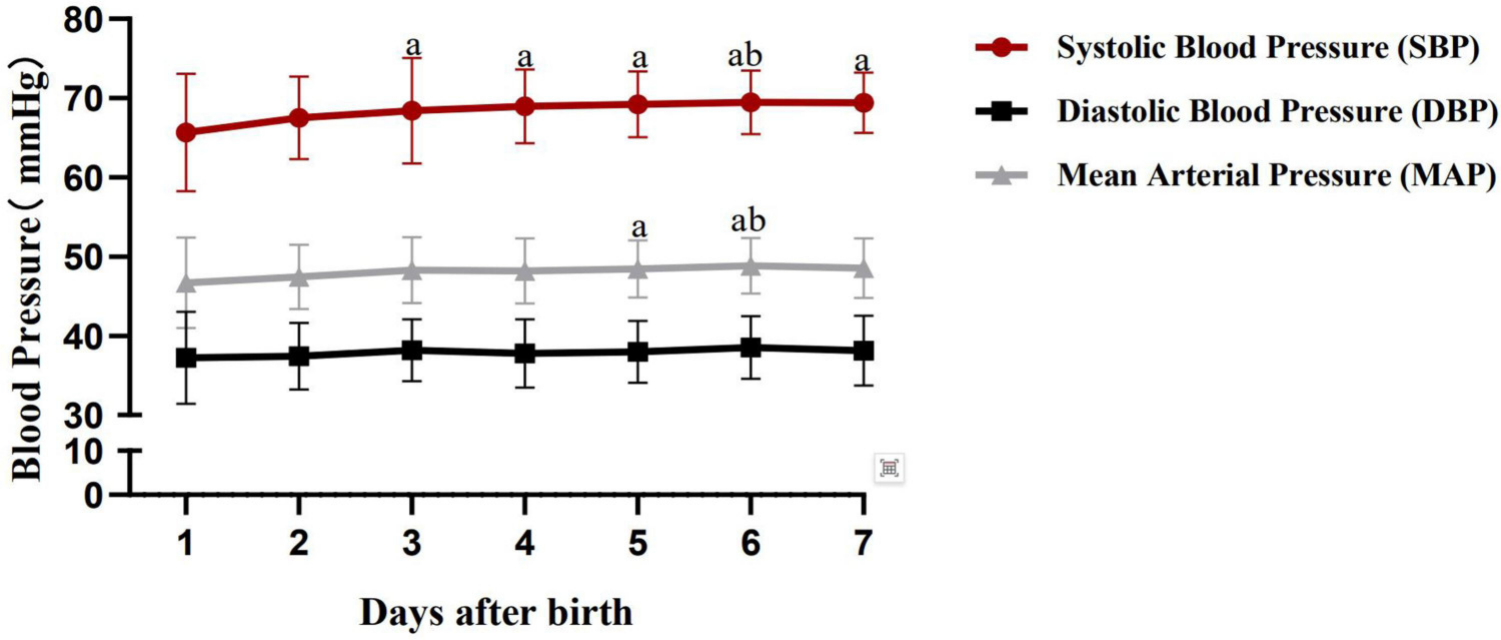
Blood pressure variation trend of preterm infants with gestational Age of 28 to <37 weeks within 7 days after birth.

The Friedman M test revealed that both systolic blood pressure (SBP) and mean arterial pressure (MAP) of preterm infants with a gestational age of 28 to <37 weeks showed an upward trend within 7 days after birth (*P* < 0.001). Further *post-hoc* pairwise comparisons revealed the following: For SBP: there were significant differences between the 1st postnatal day and the 3rd to 7th postnatal days, as well as between the 2nd postnatal day and the 6th postnatal day; no significant differences were observed between other time points. For MAP: significant differences were noted between the 1st postnatal day and the 5th to 6th postnatal days, and between the 2nd postnatal day and the 6th postnatal day; no significant differences were found between other time points. No significant variation trend was observed in diastolic blood pressure (DBP) within 1 week after birth. See Table 2 and Figure 1 for details.

**Table 2:** Blood pressure values of preterm infants with gestational Age of 28 to <37 weeks within 7 days after birth.

Time	Systolic blood pressure (SBP)	Diastolic blood pressure (DBP)	Mean arterial pressure (MAP)
1d	67.0 (61.0,70.0)	38.0 (34.0,40.0)	47.3 (43.0,50.3)
2d	69.0 (66.0,71.0)	37.0 (35.0,40.0)	47.3 (45.3,50.0)
3d	69.0 (65.0,71.0)^a^	38.0 (35.0,40.0)	48.3 (46.0,50.3)
4d	69.0 (67.0,72.0)^a^	38.0 (35.0,40.0)	48.3 (46.0,50.6)
5d	69.0 (67.0,72.0)^a^	38.0 (35.0,40.0)	48.3 (46.0,50.6)^a^
6d	70.0 (67.0,72.0)^a^^,^^b^	38.3 ± 4.0	48.6 ± 3.5^a^^,^^b^
7d	70.0 (68.0,72.0)^a^	38.0 (35.0,40.0)	48.3 (46.3,51.0)
*χ*^2^ value	51.365	11.318	28.365
P	<0.001	0.079	<0.001

a indicates *P* < 0.05 compared with the 1st postnatal day.

b indicates *P* < 0.05 compared with the 2nd postnatal day.

### 3.3 Blood pressure ranges of preterm infants with gestational Age of 28 to <37 weeks within 7 days after birth

**Figure 2:**
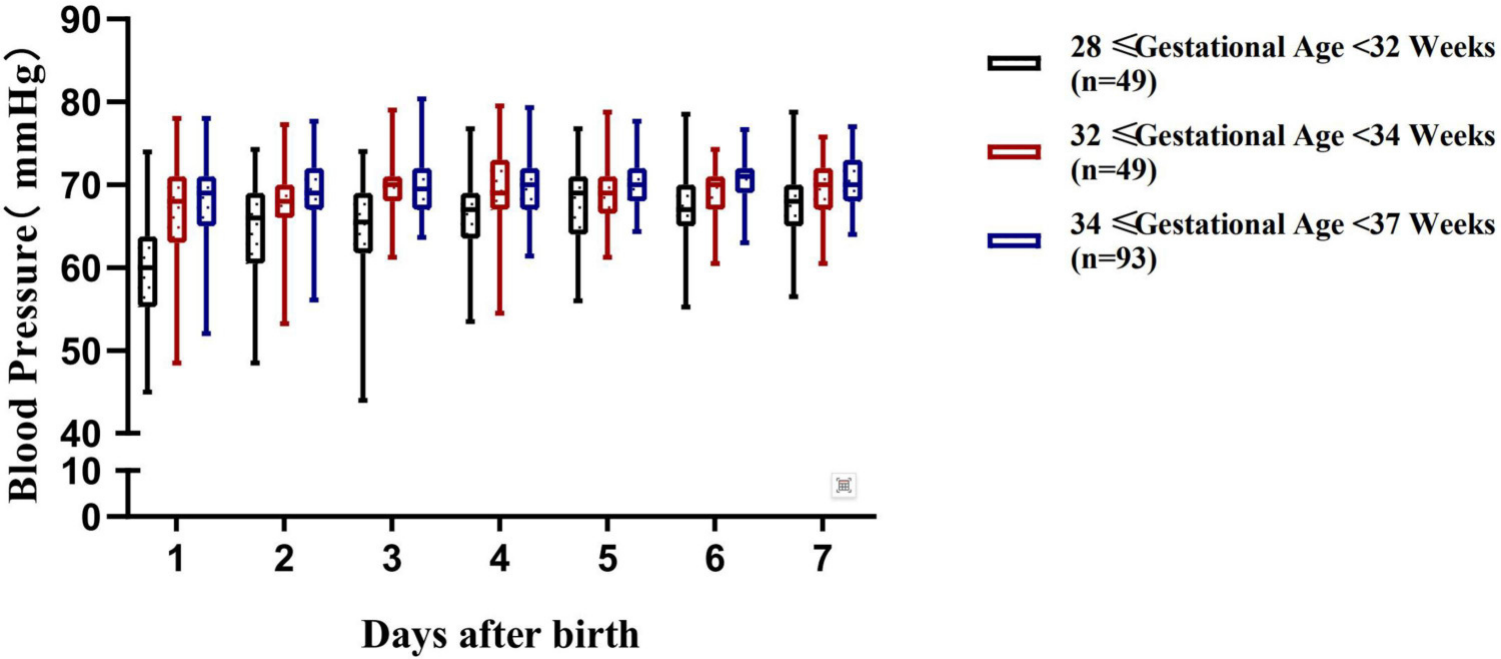
Variation range of systolic blood pressure (SBP) in preterm infants of different gestational ages within 7 days after birth.

**Figure 3:**
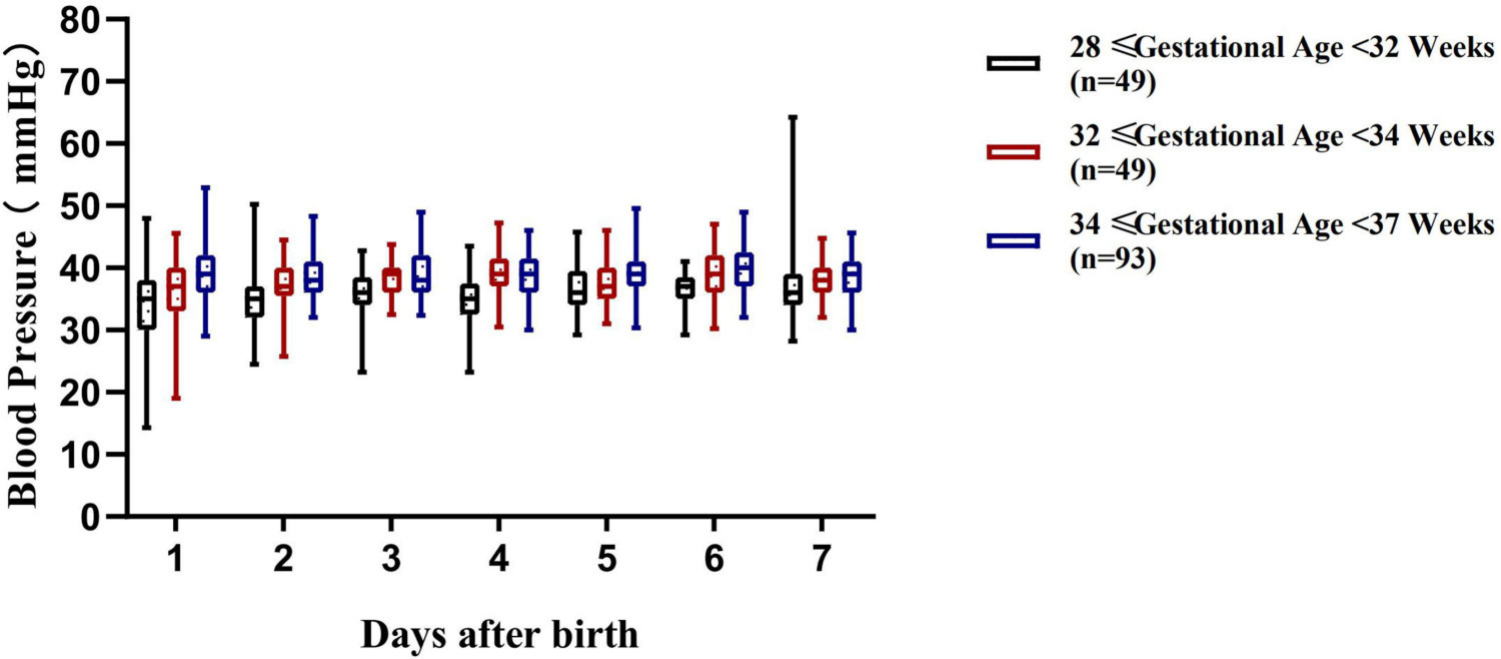
Variation range of diastolic blood pressure (DBP) in preterm infants of different gestational ages within 7 days after birth.

**Figure 4:**
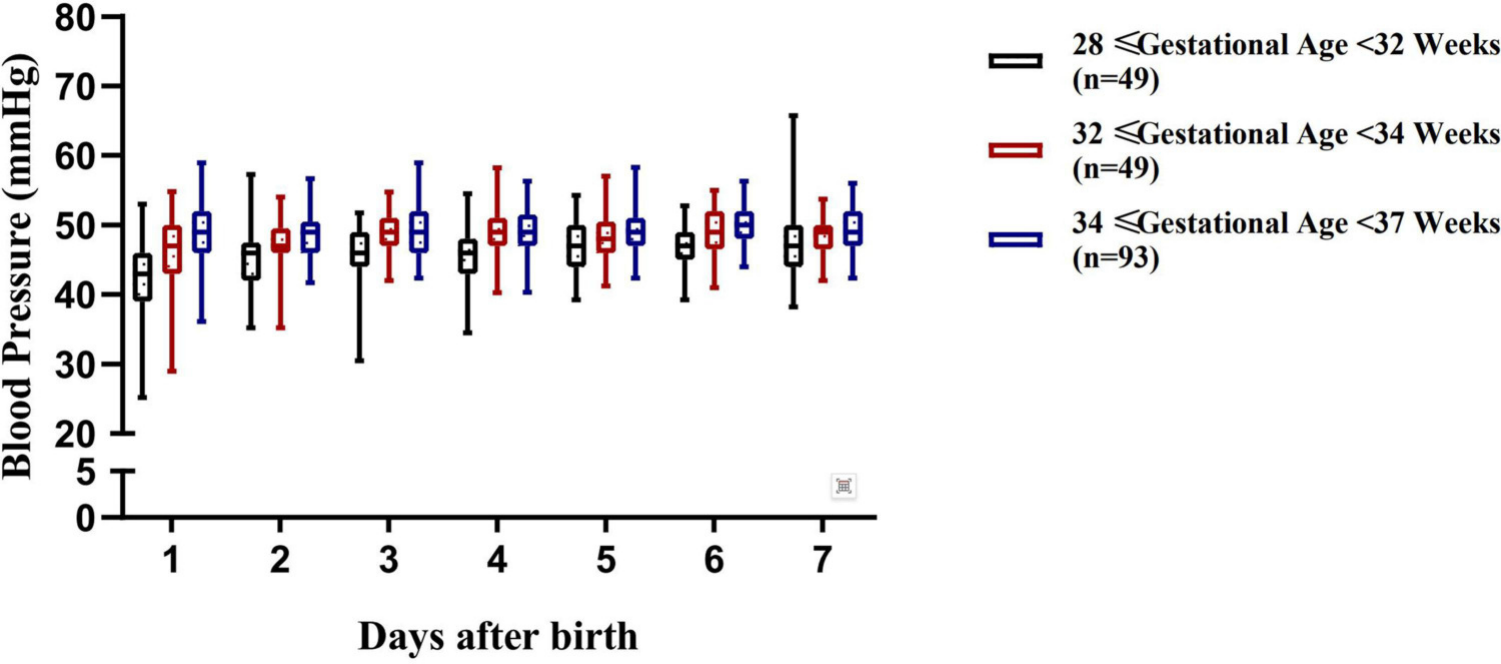
Variation range of mean arterial pressure (MAP) in preterm infants of different gestational ages within 7 days after birth.

The blood pressure ranges of preterm infants with a gestational age of 28 to <37 weeks at different time periods within 7 days after birth are shown in Table 3 and Figures 2–4.

**Table 3:** Blood pressure ranges of preterm infants of different gestational ages within 7 days after birth [mmHg, P2.5–P97.5 (P50)].

Time	28 + 0—31 + 6 Weeks (*n* = 49)			32 + 0—<33 + 6 Weeks (*n* = 49)			34 + 0—36 + 6Weeks (*n* = 93)		
	Systolic blood pressure (SBP)	Diastolic blood pressure (DBP)	Mean arterial pressure (MAP)	Systolic blood pressure (SBP)	Diastolic blood pressure (DBP)	Mean arterial pressure (MAP)	Systolic blood pressure (SBP)	Diastolic blood pressure (DBP)	Mean arterial pressure (MAP)
1d	45.0–73.9 (60.0)	14.2–47.9 (35.0)	25.4–52.6 (43.3)	48.5–78.0 (68.0)	19.0–45.6 (37.0)	28.9–55.1 (47.0)	52.0–78.0 (69.0)	29.0–52.9 (39.0)	36.4–58.9 (49.1)
2d	48.5–74.2 (66.0)	24.5–50.2 (35.0)	34.9–57.0 (45.6)	53.2–77.2 (68.0)	25.7–44.5 (37.0)	35.0–47.0 (53.9)	56.1–77.6 (69.0)	32.0–48.3 (38.0)	41.37–56.6 (48.6)
3d	44.0–73.5 (67.0)	23.2–42.7 (36.0)	30.6–51.5 (46.3)	61.2–79.0 (70.0)	32.5–43.7 (39.0)	42.1–54.7 (48.6)	61.0–80.3 (69.0)	32.3–48.9 (38.0)	42.0–59.1 (48.6)
4d	53.5–76.5 (67.0)	23.5–43.5 (35.0)	34.1–54.3 (45.6)	54.5–79.5 (69.0)	30.5–47.2 (39.0)	40.1–57.9 (49.0)	61.4–79.3 (70.0)	30.0–46.0 (39.0)	40.4–55.9 (49.3)
5d	56.0–76.7 (69.0)	29.2–45.7 (36.0)	39.1–54.4 (47.0)	61.2–78.7 (69.0)	31.0–46.0 (37.0)	41.4–56.9 (47.6)	64.3–77.6 (70.0)	30.3–49.6 (39.0)	42.5–58.1 (49.0)
6d	55.2–78.5 (67.0)	29.2–41.0 (37.0)	39.2–52.8 (46.7)	60.5–74.2 (70.0)	30.2–47.0 (39.0)	41.3–54.9 (49.3)	63.0–76.6 (71.0)	32.0–48.9 (40.0)	44.3–56.6 (50.0)
7d	56.5–78.7 (68.0)	28.5–64.2 (36.0)	38.1–65.9 (46.6)	60.5–75.7 (70.0)	32.0–44.7 (38.0)	42.0–53.8 (48.6)	64.0–77.0 (70.0)	30.0–45.6 (39.0)	42.4–55.6 (49.3)

### 3.4 Clinical outcomes and complications of preterm infants with gestational Age of 28 to <37 weeks

Among the 191 infants, one infant died following the family's decision to withdraw life-sustaining treatment due to severe (grade Ⅲ) intraventricular hemorrhage (IVH); Complications included 102 cases of bronchopulmonary dysplasia (BPD) (72 mild, 25 moderate, 5 severe), 36 cases of intraventricular hemorrhage (IVH) (19 grade Ⅰ, 14 grade Ⅱ, 3 grade Ⅲ), and 40 cases of retinopathy of prematurity (ROP) (18 stage 1, 12 stage 2, 10 stage 3).

## 4 Discussion

### 4.1 Blood pressure variation trend of preterm infants with gestational Age of 28 to <37 weeks within 7 days after birth

Normal blood pressure is defined as the vascular pressure that can maintain sufficient organ perfusion. Preterm infants undergo a transitional phase from low-resistance fetal circulation to high-resistance systemic circulation in the early postnatal period. During this period, any cause leading to decreased cardiac output or impaired adaptive changes in peripheral vascular resistance may induce hypotension (9). Currently, the commonly used definitions of hypotension include two categories: one is mean arterial pressure (MAP) lower than the gestational age value, and the other is MAP lower than 30 mmHg (10). The definition of MAP lower than the gestational age value is convenient and simple, and is most widely used clinically, but it does not consider the individual perfusion status,a increasing body of evidence has demonstrated that this definition is not reliable (11). Some scholars have found that MAP lower than 30 mmHg lasting more than 1 h is associated with severe intraventricular hemorrhage (IVH) and death, thus proposing this definition. Based on the physiological mechanism that cerebral autoregulation is impaired and perfusion decreases when the blood pressure of preterm infants is lower than 30 mmHg, is regarded as the critical closing pressure of cerebral blood vessels (12). However, this standard also has limitations, as it ignores the impact of postnatal age on the dynamic changes of blood pressure. Therefore, gestational age-specific blood pressure percentile ranges (e.g., P2.5 to P97.5) have been confirmed by multiple studies as a more reliable criterion for evaluating abnormal blood pressure in neonates.

Kent et al. found that the systolic blood pressure (SBP), diastolic blood pressure (DBP), and mean arterial pressure (MAP) of preterm infants with a gestational age of 28–37 weeks showed a rapid upward trend with increasing postnatal age within 7 days after birth, followed by stabilization (13). Consistent with this, the results of this study showed that both SBP and MAP of preterm infants in this gestational age range increased within 1 week after birth. For SBP, there were significant differences between the 1st postnatal day and the 3rd to 7th postnatal days, as well as between the 2nd postnatal day and the 6th postnatal day, with no significant differences between other time points. This suggests that SBP tends to stabilize about 3 days after birth, which may be related to the developmental law of myocardial function in preterm infants. Eriksen et al. (14) reported that the peak systolic velocity of the left ventricle gradually increases within 3 days after birth, indicating that the gradual maturation of myocardial systolic function contributes to the stabilization of blood pressure 3 days after birth. Notably, this study found no significant change in DBP within 7 days after birth, which may be related to the presence of patent ductus arteriosus (PDA) in some infants. Blood flow from the aorta shunts to the pulmonary artery through the patent ductus arteriosus, which may reduce DBP (15, 16), Although cases with large PDA were excluded in this study, small PDA may still exert potential hemodynamic effects, thereby causing certain interference with the blood pressure results. The variation trend of MAP in this study was consistent with that of SBP but with slight differences: significant differences were observed between the 1st postnatal day and the 5th to 6th postnatal days, and between the 2nd postnatal day and the 6th postnatal day, with no significant differences between other time points. This suggests that although MAP generally shows an upward trend, it stabilizes slightly later than SBP (about 6 days after birth), this discrepancy may be related to the influence of DBP on MAP. Additionally, differences between oscillometric values obtained via noninvasive monitoring and invasive arterial measurements may also exert a certain impact on MAP.

### 4.2 Blood pressure ranges of preterm infants with gestational age of 28 to <37 weeks at different time points within 7 days after birth

Although there have been numerous studies on the blood pressure characteristics of preterm infants in different gestational age subgroups, systematic and continuous observations and studies on the regularity of blood pressure changes of a broader group of preterm infants with a gestational age of 28 to <37 weeks remain relatively limited. Defining the blood pressure range for this population is of great significance for clinical management: it can not only provide precise intervention evidence for hemodynamically unstable infants, but also avoid unnecessary treatment for infants with normal blood pressure, thereby reducing excessive cardiovascular support (17). A total of 191 preterm infants with a gestational age of 28 to <37 weeks were enrolled in this study, and were stratified by gestational age. The blood pressure ranges of each subgroup at different time points within 7 days after birth were systematically established. The results showed that the regularity of blood pressure changes varying with gestational age and postnatal age in each subgroup was consistent with the study by Lalan et al. (18), further verifying that gestational age and postnatal age are core factors affecting blood pressure in preterm infants (5).

In the blood pressure assessment of preterm infants, MAP is a core reference index commonly used in clinical practice. It was previously generally believed that MAP should be roughly consistent with the infant's gestational ag**e (**19). However, this study found that the median MAP was higher than the corresponding gestational age, which was consistent with the latest study by C.J. Alonzoa et al. (20). We hypothesize that this difference may be related to the blood pressure measurement method: both this study and C.J. Alonzoa's study used non-invasive oscillometric method for blood pressure monitoring, and the measured values may be slightly higher than the true arterial pressure. In contrast, the traditional view that “MAP is consistent with gestational age” is mostly based on invasive arterial monitoring data. Invasive monitoring, while providing a closer approximation of the true blood pressure, carries risks such as infection and bleeding. Consequently, its use is largely restricted to the most critically ill infants and is not feasible for widespread application in preterm infants of 28-<37 weeks. Notably, Takci S et al. compared invasive and non-invasive readings and found good consistency between oscillometric and invasive readings (21), which makes the reference ranges established in this study clinically applicable and more likely to meet the needs of routine clinical blood pressure assessment.

In conclusion, the results of this study show that the SBP and MAP of preterm infants with a gestational age of 28 to <37 weeks increase with postnatal age within 7 days after birth, and SBP tends to stabilize about 3 days after birth. Meanwhile, the blood pressure ranges of preterm infants of different gestational ages are established, providing a reference for clinical blood pressure management. There are several limitations in this study: ①This study is a single-center retrospective observational study, which limits the generalizability of the research results. Differences in blood pressure measurement protocols, patient composition and clinical management strategies among different institutions may affect the applicability of the reference ranges in this study to other preterm infant populations. In addition, the subjects included in this retrospective study were mainly moderate and late preterm infants with relatively stable conditions who did not require immediate hypotensive intervention. The research conclusions are more applicable to this population, and caution should be exercised when extending them to critically ill preterm infants or those requiring hemodynamic intervention. ②Cases with factors that may affect blood pressure, such as patent ductus arteriosus (PDA), mechanical ventilation, and severe intraventricular hemorrhage (IVH), were not completely excluded. Although cases with large PDA were excluded, small PDA, hemodynamic changes related to mechanical ventilation, and autonomic dysregulation caused by severe IVH may still have potential impacts on blood pressure results; ③ Non-invasive oscillometric method was used for blood pressure monitoring, and the measured values were susceptible to technical operation. Although the measurement results were manually verified and the procedures were standardized, unrecognized bias may still exist. Non-invasive monitoring is clinically feasible and suitable for daily clinical practice, Although its results can serve as routine clinical references, they are not equivalent to absolute values of invasive blood pressure. The blood pressure data proposed in this study are descriptive and exploratory, and not intended as authoritative clinical normative standards. Future multicenter, prospective studies will be conducted to expand the sample size, optimize exclusion criteria, further control hemodynamic-related confounding factors, and elevate the level of evidence-based support for blood pressure management in preterm infants.

## Data Availability

The original contributions presented in the study are included in the article/Supplementary Material, further inquiries can be directed to the corresponding author.
